# Prognostic Value of SPARC in Patients with Pancreatic Cancer: A Systematic Review and Meta-Analysis

**DOI:** 10.1371/journal.pone.0145803

**Published:** 2016-01-05

**Authors:** Wei Han, Fang Cao, Min-bin Chen, Rong-zhu Lu, Hua-bing Wang, Min Yu, Chun-tao Shi, Hou-zhong Ding

**Affiliations:** 1 Department of General Surgery, Kunshan First People's Hospital Affiliated to Jiangsu University, Kunshan Jiangsu, 215300, P. R. China; 2 Department of Radiotherapy and Oncology, Kunshan First People's Hospital Affiliated to Jiangsu University, Kunshan Jiangsu, 215300, P. R. China; 3 Department of Preventive Medicine, School of Medicine, Jiangsu University, Zhenjiang Jiangsu, 212001, P. R. China; University of Nebraska Medical Center, UNITED STATES

## Abstract

**Objective:**

There is a heated debate on whether the prognostic value of SPARC is favorable or unfavorable. Thus, we carried out a meta-analysis evaluating the relationship between SPARC expression and the prognosis of patients with pancreatic cancer.

**Methods:**

We searched PubMed, EMBASE and Web of Science for relevant articles. The pooled hazard ratios (HRs) and corresponding 95%CI of overall survival (OS) were calculated to evaluate the prognostic value of SPARC expression in patients with pancreatic cancer. We also performed subgroup analyses.

**Results:**

With 1623 patients pooled from 10 available studies, the incorporative HR showed an unfavorable prognosis of patients with pancreatic cancer in the multivariate analysis (HR = 1.55, 95%CI: 1.11–2.17, P = 0.01), but not in univariate analysis (HR = 1.41, 95%CI: 0.47–4.21, P = 0.54) and estimate (HR = 1.24, 95%CI: 0.72–2.13, P = 0.44). And this adverse impact could also be found in the subgroup analyses in multivariate analysis, especially in the stroma (HR = 1.53, 95%CI: 1.05–2.24, P = 0.03). However, the combined HR had the highly significant heterogeneity. No obvious publication bias was found.

**Conclusions:**

SPARC might be an unfavorable indicator in patients with pancreatic cancer, especially in the stroma. More and further researches should be conducted to reveal the prognostic value of SPARC.

## Introduction

In spite of the advanced resection and chemoradiotherapy, pancreatic cancer, with the worst prognosis, has become a global challenge[1]. And considering that the overall one-year and 5-year survival rate are only 27% and 6%, respectively[2], it is especially crucial to search ways for early diagnosis, effective therapy and preventing recurrence. The inspection of CEA and CA19-9 can improve the prognosis in postoperative patients with digestive system neoplasms, including pancreatic cancer[3]. However, not like other digestive system neoplasms, pancreatic cancer is lack of specific biomarkers. Thus, a huge bulk of studies have been performed to identify potential biomarkers, in order to improve survival of patients with pancreatic cancer.

Secreted protein acidic and rich in cysteine (SPARC), also termed osteonectin or BM-40, one of the eight members of the SPARC family, which are invovled in human development and disease[4], is a 32–35 kDa multifunctional collagen or calcium-binding ECM glycoprotein located at 5q33.1 and is a single polypeptide with 285 amino acids including three biological structural domains, the acidic N-terminal (NT) domain, a follistatin-like domain and a Ca2+ binding extracellular domain[5–7].

In human carcinogenesis, SPARC plays remarkable roles in altering the activity and the microenvironment of cancer cells, modulating cell growth, apoptosis, adhesion migration and invasion, regulating ECM and the activity of matrix metalloproteinases[4,8]. Hence, there are many studies evaluating the effect of SPARC expression on digestive systems neoplasms. Downregulating of SPARC by siRNA-mediated knocking down its gene inhibits growth and invasion of MGC803 and HGC27 gastric cancer cells[9]. In colorectal cancer, SPARC expression in MSC (HR, 0.654; CI,0.409–1.048; p = 0.028; HR, 0.536; CI, 0.359–0.802; p = 0.002) were all independent prognostic factors for OS and DFS, respectively, and the low expression of SPARC was also related with the high level of TNM[10]. Thus, reduced expression of SPARC is associated with poor prognosis and aggressive clinicopathological features in both cancer cells and MSC[10]. Further more, a meta-analysis[11] indicated the negative prognostic value of SPARC expression in patients with gastric cancer, with the relative risk of OS (RR = 1.78, 95% CI: 1.52–2.09, Z = 7.10, p = 0.43). And in pancreatic cancer, the results from published reports about the prognostic value of SPARC are also controversial. Guweidhi[12], reported that a 31-fold increase in osteonectin mRNA levels in PDAC as compared with the normal pancreas (P<0.01), and in metastatic tissues, strong immunoreactivity was observed in fibroblasts and in extracellular matrix surrounding metastatic cancer cells, whereas the signal was absent in most tumor cells. So, SPARC overexpressed in pancreatic cancer has the potential to improve the invasiveness of cancer cells[12]. Puolakkainen[13] examined the growth of pancreatic tumors in SPARC-null (SP(-/-)) mice and their wild-type (SP(+/+)) counterparts. And found that the growth of pancreatic tumors in SPARC-null mice were enhanced because of the collagen deposition and fiber formation decreasing[13]. Compared to in the tumor cells, where expression of SPARC is commonly downregulated by promoter methylation, overexpression of SPARC is frequently found in the stroma[14].

In view of the heated controversy of SPARC, a systematic review of the available articles with meta-analysis is urgent to be performed to evaluate the prognostic value of SPARC in pancreatic cancer.

## Materials and Methods

### Database search strategy

We performed systematic literature search of Pubmed, EMBASE and Web of Science from their incipiency to August, 2015. The retrieval strategy was used as follow: (SPARC or "Secreted protein acidic and rich in cysteines" or osteonectin or BM-40) AND (pancreatic or pancreas*) AND (cancer or tumor or tumour or neoplasm or carcinoma or adenocarcinoma) AND (prognosis or prognostic or predict or survival or outcome or prognos*). Reference lists of articles and reviews were hand-searched for additional studies. Manuscripts were also manually scanned to obtain potential articles most relevant to this review. Only studies published in peer reviewed journals were included. All articles were written in English. All the initially identified articles were scrutinized independently by two reviewers (Han W and Cao F). There was no protocol developed for this review.

### Inclusion criteria

To be eligible for inclusion, the following criteria had to be fulfilled: (a) clinical studies researched patients with pancreatic cancer; (b) SPARC expression in pancreatic cancer was measured with methods such as immunohistochemistry (IHC) or Quantitative real-time polymerase chain reaction (qRT-PCR); (c) studies reported the association between SPARC expression and survival outcome; (d) studies contained HRs and 95% CI for OS according to SPARC status which either were reported or could be estimated from the relevant published data[15]; (e) only the most recent report or the most integrated report would be enrolled, if the study population was duplicated or overlapping. Disagreement was resolved by discussion between the two reviewers or consultation with a third reviewer (Chen MB).

### Exclusion criteria

Exclusion criteria were: (a) literature published as letters, editorials, abstracts, reviews, case reports and expert opinions; (b) experiment in vitro or in vivo but not based on patients; (c) articles without the HRs with 95% CI about overall survival, or the K-M survival curves; (d) repeated and similar studies.

### Data extraction

The following information from each article was extracted: (a) general information, including first author, publication year, country (area) of origin, age and gender of the study patients, sample size and the follow-up duration; (b) method to determine SPARC expression and number of patients stratified by SPARC expression; (c) clinical outcomes, including OS or DFS and its correlative HRs with 95%CI. When an article only had K-M curves,we used Engauge Digitizer, a digitizing program, that could translate curves into numbers to extract survival data from its curves, and then put the data into a spreadsheet, called Tierney table, by which the estimated HR and corresponding 95%CI were calculated immediately[16].

### Quality assessment

The two independent reviewers (Han W and Cao F) assessed the quality of each study with the Newcastle-Ottawa Quality Assessment Scale (NOS)[17]. This scale mainly used in non-RCT studies. We used the quality assessment scale of cohort studies. A study with NOS >5 was regarded as a high-quality study[18]. Disparity was resolved by discussion or consultation.

### Data synthesis and analysis

The primary outcome was OS associated with SPARC expression in patients with pancreatic cancer. HR and 95% CI were used to be the effect measure of interest. A combined HR>1, with its 95% CI did not overlap 1, indicated a worse survival for the group with high SPARC expression. The heterogeneity among studies was measured using the Q and I^2^ test. A random or Fixed model was used according the heterogeneity analysis. A random effect model was applied if I^2^≧50%; the fixed effect model was selected if I^2^<50%. When I^2^≧50%, subgroup analyses would be carried out. A P < 0.05 indicates a significant factor contributing to the observed heterogeneity. The latent publication bias was assessed by a funnel plot and Egger’s linear regression test, and a value <0.05 indicated an inevitable significant publication bias[19]. All statistical tests were two-tailed and P<0.05 was considered statistically significant. All the analyses were conducted by Review Manager software version 5.3 (The Cochrane Collaboration) and STATA statistical software package version 12.0 (Stata Corporation, College Station, TX).

## Results

### Search results

**Fig 1:**
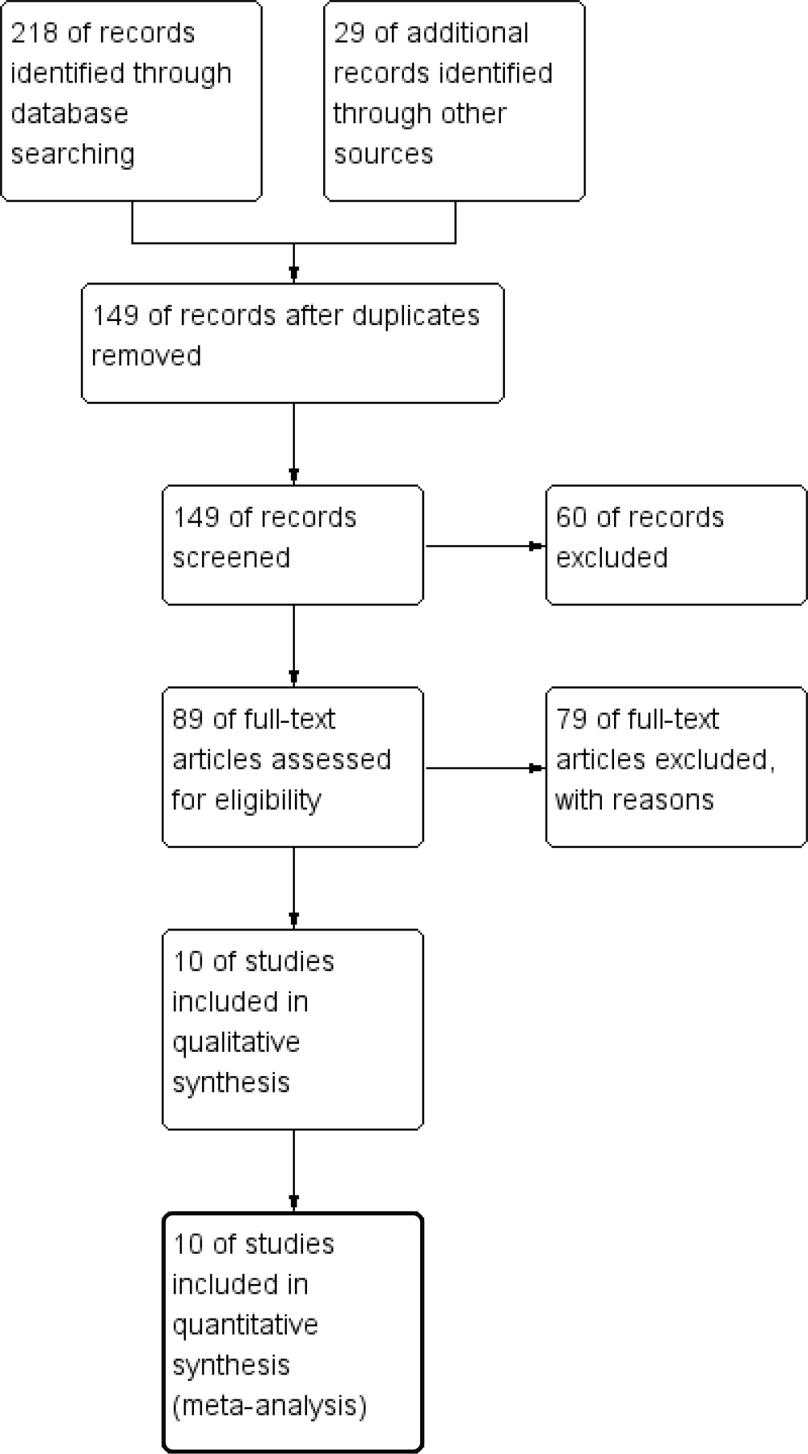
Flow chart for the selection of records to include.

A total of 218 articles were retrieved in the initial search of databases. In addition, 29 records were yielded by manual searching. After removing 98 duplicates, we read the titles and abstracts of the 149 studies left. 60 citations were excluded from analysis based upon abstracts or titles, leaving 89 studies for further full-text review. At the same time, one study could't be found anyway[20]. After meticulously reading, 78 studies were excluded: 76 studies, including reviews, were excluded because of no or insufficient survival data; and the two left were excluded in that their survival datum were only about the methylation of SPARC gene and the overexpression of SPARC in patients with ampullary cancer, respectively[21,22]. As a result, 10 eligible studies[23–32] with 1632 patients in total, were enrolled in this meta analysis (Fig 1).

### Study characteristics and quality assessment

The basic characteristics of the 10 studies are summarized in Table 1 and Table 2. Briefly, study sample sizes ranged from 31 to 557; 8 studies were conducted in Caucasian populations, while the remaining used Asian populations[27,28]. Patients in 4 of the 10 cohorts focused on the advanced or metastatic pancreatic cancer[24,26,29,32], and patients in 6 of the 10 received surgical operation as the main treatment[23,25,27,28,30,31]. All studies investigated SPARC in cancer cells or stromal cells of tumor tissues by IHC or RT-PCR. Mouse monoclonal antibody were used in IHC, but two studies didn’t illustrate what kind of antibodies they used[27,32]. Two studies used both of the two methods but only used IHC to identify the cut-off[27,32]. Additionally, the stratification of SPARC expression varies among studies. In IHC measured studies, three different methods were used to determine the SPARC cut-off values: extent, intensity and both of them. Although each of these studies was an arbitrary cut-off seemingly, the extent ranges from 10% to 25%, and intensity is about "+", or its score is about "1"; and none of them had a cutoff more than 30% or “++”. So, they all had a cutoff low to intermediate. In the two studies[28,30] measured by qRT-PCR, their cut-off were 4.3 and 1 respectively which were the median values. In five studies which investigated SPARC both in the tumor and the stroma[24,25,27,31,32], one study only presented one result with the multivariate analysis in the stroma[24], one only presented one Kaplan-Meier curve of SPARC expression in the tumor[27] and another presented a Kaplan-Meier curve without differentiating in the stroma or in the tumor[32]. HRs with 95% CIs of OS and DFS reported or estimated, were also listed in Table 2. Five of these eligible articles considered SPARC as an indicator of poor prognosis, while three revealed no significant impact on overall survival[24,29,30]. Two reported SPARC as an indicator of good prognosis[27,32]. And of the five articles which reported the poor prognostic value of SPARC, one reported that SPARC in the stroma, but not in the tumor, was associated with worse survival[25]. Another which considered SPARC in the stroma, but not in the tumor was related to better outcome was also reported[32]. Furthermore, Hidalgo[24] had three results, one multivariate analysis, the other two univariate, and Infante[25] and Sinn[31] both had two results in the multivariate analysis and in the estimate, respectively.

**Table 1:** Main characteristics of all the studies included in the meta-analysis.

First author	Year	Study region	Sample	NOS	Age (years)	N.of M/F	Primary antibody	Treatment	Follow-up(months)
Gundewar[23]	2015	Sweden	Tissue	7	66(48–84)	43/45	mouse mAb	surgery	about 10 years
Hidalgo[24]	2015	Spain	Tissue	6	NR	NR	mouse mAb	chemotherapy	>30
Infante[25]	2007	USA	Tissue	8	67	NR	mouse mAb	surgery	>48
Mantoni[26]	2008	UK	Tissue	7	NR	27/22	mouse mAb	CRT	>70
Mao[27]	2014	China	Tissue	6	<60:72	86/64	mAb	surgery	>36
					≥60:78				
Miyoshi[28]	2010	Japan	Tissue	8	66(36–86)	66/38	-	surgery	median20(1–101)
Ormanns[29]	2015	Germany	Tissue	6	62	NR	mouse mAb	chemotherapy	NR
Prenzel[30]	2006	Germany	Tissue	7	59.4(33–81)	24/15	-	surgery	median9.5
Sinn[31]	2014	Germany	Tissue	7	62(36–81)	65/95	mouse mAb	surgery	>100
Von Hoff[32]	2011	USA	Tissue	6	61.7(28–86)	32/35	mAb and pAb	chemotherapy	>20

N. Of P.: the number of patients; mAb: monoclonal antibody; pAb: polyclonal antibody; NR: Not reported; CRT: chemoradiotherapy; Mao and Von Hoff didn’t explain what kind of Ab, so we consider them as other Abs; In the column of age, for example, 66(48–84) means mean (ranges).

**Table 2:** Main characteristics of all the studies included in the meta-analysis.

First author	N. of P.	Method	cut-off of SPARC high expression	Cell type /Location	Outcome	HR obtainment	HR	95%CI
Gundewar[23]	88	IHC	E > 10%& I > 1	Stroma	OS	Reported (M)	2.12	1.19–3.98
Hidalgo[24]	131	IHC	E: IHC score of >2	Stroma	OS	Reported (M)	1.395	0.904–2.153
	125			Stroma	OS	Reported (U)	0.658	0.423–1.023
	301			Tumor	OS	Reported (U)	1.16	0.52–2.62
Infante[25]	299	IHC	E≥10%& I>+	Stroma	OS	Reported (M)	1.89	1.31–2.74
				Tumor	OS	Reported (M)	1.02	0.73–1.42
Mantoni[26]	73	IHC	E*I ≥ 1	Stroma	OS	Reported (M)	2.23	1.05–4.72
Mao[27]	150	IHC	E: with weak	Tumor	OS	Estimated	0.59	0.356–0.978
		RT-PCR	or focal labeling					
Miyoshi[28]	104	qRT-PCR	value >4.3	-	OS	Reported (M)	2.918	1.629–5.504
					OS	Reported (U)	3.815	1.737–7.480
Ormanns[29]	134	IHC	E ≥ 25%	Stroma	OS	Reported (M)	0.83	0.56–1.21
Prenzel[30]	31	qRT-PCR	T value>1	-	OS	Estimated	2.94	0.422–20.466
Sinn[31]	160	IHC	I: A four-tier	Stroma	OS	Estimated	1.41	1.008–1.984
			scoring system;		DFS	Reported (M)	1.47	1.02–2.14
			EI: IRS≥3	Tumor	OS	Estimated	2.03	1.372–3.004
					DFS	Reported (M)	1.61	1.07–2.40
Von Hoff[32]	36	qRT-PCR	average z-scores≥0	Stroma &	OS	Estimated	0.81	0.189–3.47
		IHC	(EI)	Tumor				

N. Of P.: the number of patients; OS: overall survival; DFS: disease-free survival; HR: hazard ratio; “M”: the multivariate analysis; “U”: the univariate analysis; “E”: identifying the cut-off by the extent; “I”: identifying the cut-off by the intensity.

None of these ten studies gained a NOS <6, suggesting that all of them had high levels of methodological quality in this meta-analysis (Table 3).

**Table 3:** Quality assessment of eligible studies with Newcastle-Ottawa Scale.

First author	Year	NOS	Selection	Comparability	Outcome
Gundewar[23]	2015	7	★★★*	★★*	★★
Hidalgo[24]	2015	6	★★★	★	★★*
Infante[25]	2007	8	★★★	★★	★★★
Mantoni[26]	2008	7	★★★	★★*	★★
Mao[27]	2014	6	★★★*	★	★★
Miyoshi[28]	2010	8	★★★	★★	★★★
Ormanns[29]	2015	6	★★★*	★	★★
Prenzel[30]	2006	7	★★★	★★	★★
Sinn[31]	2014	7	★★★	★★	★★*
Von Hoff[32]	2011	6	★★★*	★*	★★

* The score was produced by discussion.

### SPARC and overall survival

**Fig 2:**
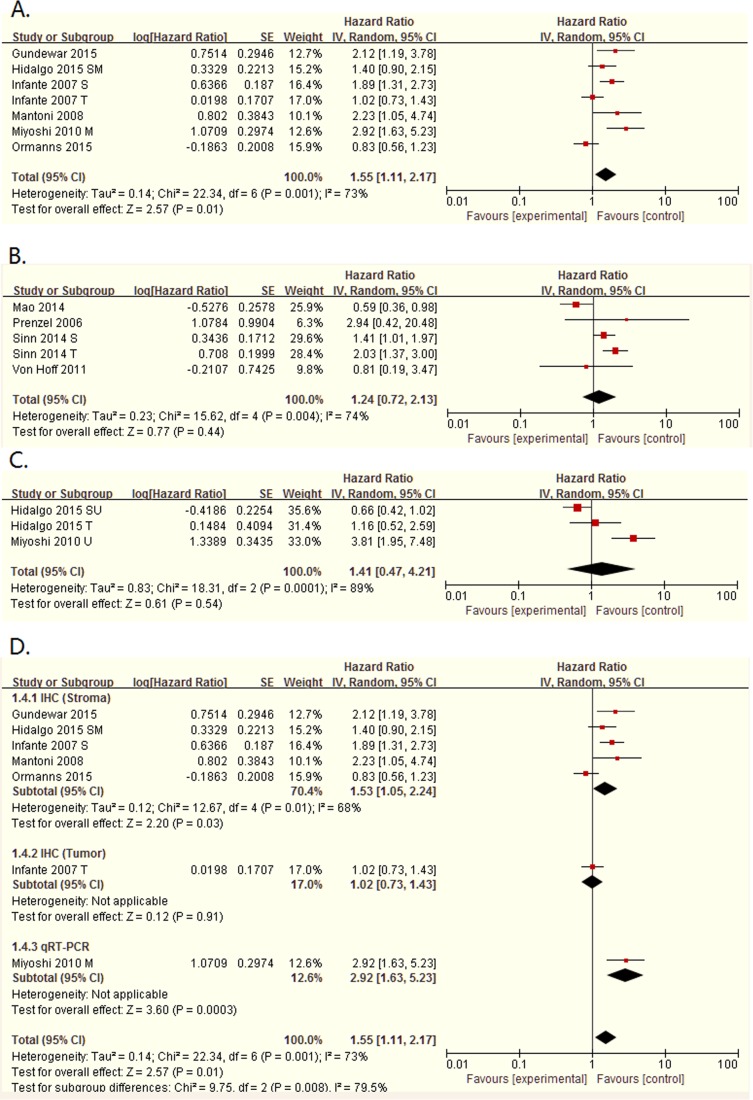
Forest plot of hazard ratios (HRs) for OS of high SPARC expression versus low expression in pancreatic cancer. A. The HRs for OS in multivariate analysis; B. The HRs for OS in Estimate; C.The HRs for OS in univariate analysis; D. The subgroup about detection methods, including in the stroma and in the tumor, in the multivariate analysis.

All of these ten studies showed the association between SPARC expression and overall survival of patients with pancreatic cancer. In spite of highly significant heterogeneity (Tau² = 0.14; Chi² = 22.34, P = 0.001; I² = 73%), the pooled HR for all of these seven studies with multivariate analysis was 1.55 (95%CI: 1.11–2.17, P = 0.01. Table 4, Fig 2), illustrating that elevated SPARC expression was significantly related with poor OS of patients with pancreatic cancer. However, the two pooled HRs of the studies with univariate analysis and estimate, both had no significance in statistic (P = 0.54 and P = 0.44, respectively).

**Table 4:** Subgroup analyses of multivariate analysis and estimate for overall survival.

		Multivariate analysis					Estimate			
	N	HR (95%CI)	I²	Ph	P	N	HR (95%CI)	I²	Ph	P
Overall	7	1.55 [1.11, 2.17]	73%	0.001	0.01	5	1.24 [0.72, 2.13]	74%	0.004	0.44
Cell type/Location										
Stromal	5	1.53 [1.05, 2.24]	68%	0.01	0.03	1	1.41 [1.01, 1.97]	-	-	0.04
Tumor	1	1.02 [0.73, 1.43]	-	-	0.91	2	1.11 [0.33, 3.71]	93%	0.0002	0.87
Stroma&Tumor	0	-	-	-	-	1	0.81 [0.19, 3.47]	-	-	0.78
Treatment										
Surgical	4	1.79 [1.13, 2.83]	76%	0.005	0.01	4	1.30 [0.72, 2.35]	80%	0.002	0.39
Non-surgical	3	1.28 [0.76, 2.15]	69%	0.04	0.35	1	0.81 [0.19, 3.47]	-	-	0.78
Study region										
Asian	1	2.92 [1.63, 5.23]	-	-	0.0003	1	0.59 [0.36, 0.98]	-	-	0.04
Caucasian	6	1.41 [1.02, 1.94]	68%	0.007	0.04	4	1.63 [1.25, 2.13]	5%	0.37	0.0003
Sample size										
<100	2	2.16 [1.37, 3.42]	0%	0.92	0.001	2	1.31 [0.39, 4.43]	8%	0.30	0.67
≥100	5	1.41 [0.95, 2.09]	78%	0.001	0.09	3	1.22 [0.65, 2.29]	86%	0.0007	0.54
Detection method										
IHC	6	1.49 [1.04, 2.14]	76%	0.0009	0.03	4	1.17 [0.66, 2.07]	80%	0.002	0.60
qRT-PCR	1	2.23 [1.05, 4.74]	-	-	0.04	1	2.94 [0.42, 20.48]	-	-	0.28
Scoring method										
E	2	1.07 [0.64, 1.77]	67%	0.08	0.80	1	0.59 [0.36, 0.98]	-	-	0.04
I	-	-	-	-	-	1	1.41 [1.01, 1.97]	-	-	0.04
EI	4	1.99 [1.50, 2.65]	67%	0.03	0.02	2	1.69 [0.83, 3.47]	30%	0.23	0.15

In view of the heterogeneity, we conducted the subgroup analyses, presented in Table 4, by stratifying the pooled data according to cell type/location (stromal cells vs. tumor cells or in the stroma vs. in the tumor), main treatment (surgical vs. non-surgical), study region (Asian vs. Caucasian), sample size (<100 vs. ≥100), detection method (IHC vs. qRT-PCR) and scoring method (E vs. I vs. EI). From this table, in the multivariate analysis, we could found that SPARC in the stroma was related to poor survival with a pooling HR being 1.53 (95%CI: 1.05–2.24, P = 0.03), but also with a high heterogeneity (I² = 68%, Ph = 0.01). However, we cannot get the pooled HR in the tumor, because of the only one study in multivariate analysis. And in the estimate HRs, two studies about the expression in tumor were pooled, but had no significance (HR = 1.11, 95%CI: 0.33–3.71, P = 0.87). There were four studies treated by surgery in the multivariate analysis, with a pooled HR = 1.79 (95%CI: 1.13–2.83, P = 0.01) and a high heterogeneity (I^2^ = 76%, Ph = 0.005). The left three studies were treated non-surgery, but their pooled HR had no significance (P = 0.35). Also, in the estimate, the pooled HR of surgical had no significance (P = 0.39). In the region of Caucasian, we can see that both the multivariate analysis and the estimate had significant pooled HR (HR = 1.41, 95%CI: 1.02–1.94, P = 0.04; HR = 1.63, 95%CI: 1.25–2.13, P = 0.0003), and the estimate had a low heterogeneity with I2 = 5%. Then, we performed the subgroup analysis demixed by sample size, and I²being 0 and 8% were found in the studies with size <100, while the subgroup of the studies with size ≥100 still had heterogeneity. And only found a P<0.05 in the multivariate analysis (HR = 2.16, 95%CI: 1.37–3.42, P = 0.001), but not in the estimate. Thus, sample size might be the source of heterogeneity. As for the detection method and the scoring method, only found a pooled HR in IHC, and another one in EI had a P<0.05, still with a high heterogeneity. None of the other had no significance. In the univariate analysis, two were detected by IHC, but reported the HRs in the stroma and in the tumor, respectively, and the other was detected by qRT-PCR. Also, they all had a sample size>100. So, we didn’t carry out a subgroup analysis in the univariate analysis.

Due to only one study[31], we failed to calculate the pooled HR of DFS.

### Sensitivity analyses

To value the stability of our results, a sensitivity analysis was performed. In the multivariate analysis and the univariate analysis, no significant changes were detected between the previous and new HRs, the latter pooled by the studies left when we deleted an individual study at a time. However, in the estimate, when deleting the study of Mao[27], we found that a new pooled significant HR = 1.63 (95%CI: 1.25–2.13, P = 0.0003), which supported the negetive prognostic value of SPARC expression in patients with pancreatic cancer, as compared with the old pooled HR with a P = 0.44. Different study region may be the reason just as the results in subgroup analysis (Fig 2B and S2 Fig). In addition, it is noted that two studies both had two results, so we displayed the S3 Fig to prove this stability.

### Publication bias

**Fig 3:**
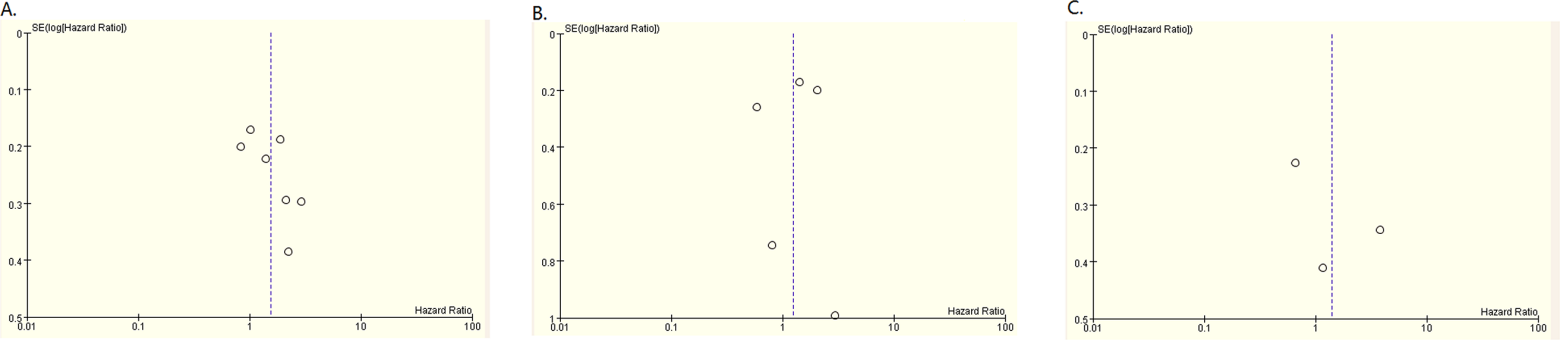
Funnel plot of 10 studies. A. was about multivariate analysis; B. was about estimate; C. was about univariate analysis.

Due to ten studies, we carried out the publication bias assessment of the ten eligible studies. There was no obvious asymmetry in these funnel plots (Fig 3), multivariate analysis, estimate and univariate analysis. And no evident publication bias was found, with the P value of Egger’s test (P = 0.117, P = 0.819 and P = 0.531, respectively).

## Discussion

SPARC, as a potential molecular marker of prognosis in malignant tumors, has generated remarkable interest in this crucial period of the high morbidity and mortality of malignancies. However, there is a heated controversy whether high or low SPARC expression is correlated with poor survival. SPARC expression is an unfavorable outcome in gastric, prostate and lung cancers [33,34,35], while as a favorable prognostic indicator, SPARC has been reported for colorectal cancer[36]. In pancreatic cancer, many studies have investigated the prognostic value of SPARC, in spite of small sample sizes and controversial reports. And some of them considered a close relationship between SPARC expression and survival after treatment regimens including nab-paclitaxel and gemcitabine[32]. In addition, no meta-analyses have formerly been performed on the prognostic significance of SPARC in pancreatic cancer. To clarify this question and explore its role in the prognosis of pancreatic cancer, we carried out a meta-analysis.

As we all know, this meta-analysis, with a total 10 studies and 1632 patients, was the first systematic review which evaluated the role of SPARC in the prognosis of pancreatic cancer. The pooled HR of OS indicated that high expression of SPARC had a poor survival in patients with pancreatic cancer. This conclusion could be showed in subgroup analyses, especially in the subgroup of cell type, which showed that high SPARC expression in the stroma, but not in the tumor, was a strong prognostic indicator of lower OS. In addition, with their I² = 0, the sample size < 100, may be the sources of the heterogeneity. Furthermore, the funnel plots were established with no dissymmetry and a Egger’s test was performed gaining a P>0.05 in multivariate analysis, univariate analysis and estimate, indicating that our results were robust.

SPARC is useful for the adjuvant chemotherapy of pancreatic cancer. Nab-paclitaxel (Nab-P) is active in pancreatic cancer refractory to gemcitabine[37] and the combination of nab-paclitaxel and gemcitabine is safe and effective for patients with metastatic pancreatic cancer[38]. According to three different clinical trials including melanoma, pancreatic, and neoadjuvant breast cancer, SPARC was considered as a predictive biomarker of response to nab-paclitaxel[39]. However, in our subgroup analysis, we only found that the pooled HR of surgical in multivariate analysis was significant in statistic. Maybe, more studies should be carried out for further exploration.

With the most research and the deepest exploration among all of the members of this family, SPARC has been found to have many significant molecular mechanisms in malignancies, including modulating ECM and the tumor microenvironment (TME), anti-adhesive and regulating apoptosis, tumor growth, migration and invasion[40]. SPARC was also involved in stirring up the activation of TGFβ[41], regulating the Notch1/STAT3[42] and the p53/p21Cip1/ Waf1[43] pathway and enhancing the response to chemotherapy and radiation[44,45]. And the expression of KLF4, a tumor suppressor, could inhibit SPARC expression to restrain the tumor invasion[46]. These theories supported the poor survival associated with elevated SPARC expression.

In this meta-analysis, there were some limitations. Firstly, the sample size was still small. In spite of 1632 patients in total, most of whom come from healthcare centers or hospitals with sufficient follow-ups, there were only 829 patients pooled in the multivariate analysis of overall survival. Secondly, the HRs estimated from Kaplan-Meier curves were imprecise. These might influence our consequence. Thirdly, because most of studies were retrospective, selection bias, information bias and other biases were inevitable. Fourthly, none of these eligible studies divided patients into two groups by TNM stage. However, in another study investigating SPARC expression in patients with esophageal squamous cell carcinoma (SCC), the author evaluated the relationship between SPARC expression in the stroma and overall survival through two groups, stage-IIA/IIB and stage-III/IV esophageal SCC[47]. And the result indicated that patients in stage-IIA/IIB, but not in stage-III/IV esophageal SCC with high SPARC expression, had a poor survival. Thus, patients in different stages might affect our results, including the heterogeneity. Finally, we only searched the studies in English. This could lose some available studies in other languages. And some unpublished studies could also be ignored. Thus, our results might be flawed, to some extent.

## Conclusions

In this systematic review with meta-analysis, the elevated SPARC expression, especially in the stroma, was associated with a poor prognosis in patients with pancreatic cancer. Further studies should be performed to confirm our conclusion and explore its molecular functions.

## Supporting Information

S1 PRISMA Checklist PRISMA checklist. (DOC)

S1 Fig Egger’s test. (DOC)

S2 Fig Sensitivity analyses of estimate without the study of Mao. (DOCX)

S3 Fig Sensitivity analyses of multivariate analysis and estimate. (DOCX)

S1 Table Clinical Studies Checklist. (DOCX)
