# Supplementary figures and images for: Prognostic Value of SPARC in Patients with Pancreatic Cancer: A Systematic Review and Meta-Analysis
Source: PLoS One. 2016 Jan 5;11(1):e0145803. doi: 10.1371/journal.pone.0145803 (PMC4701416; doi:10.1371/journal.pone.0145803)

Multivariate:


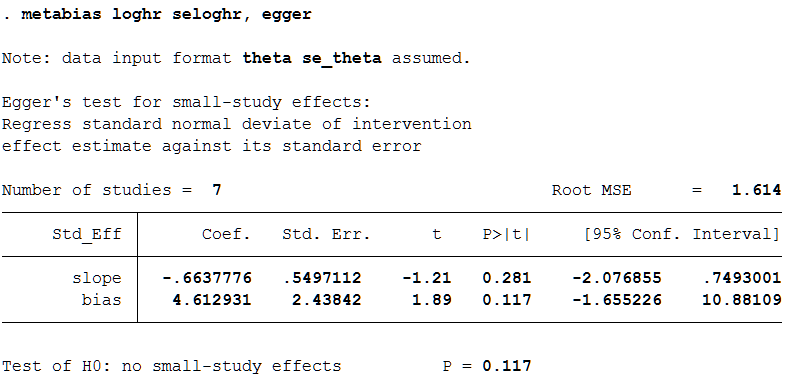


Estimate:


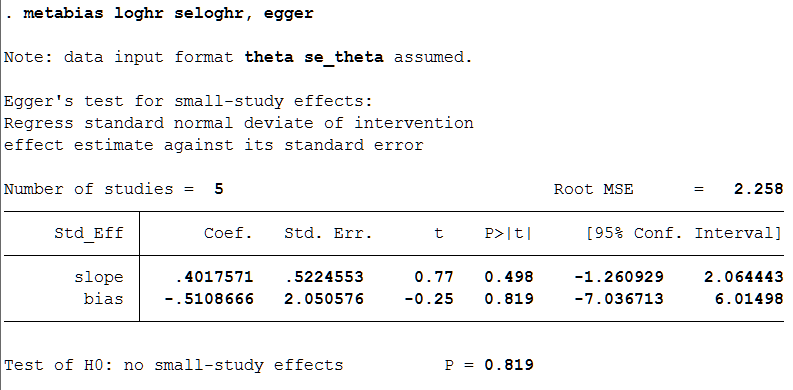


Univariate:


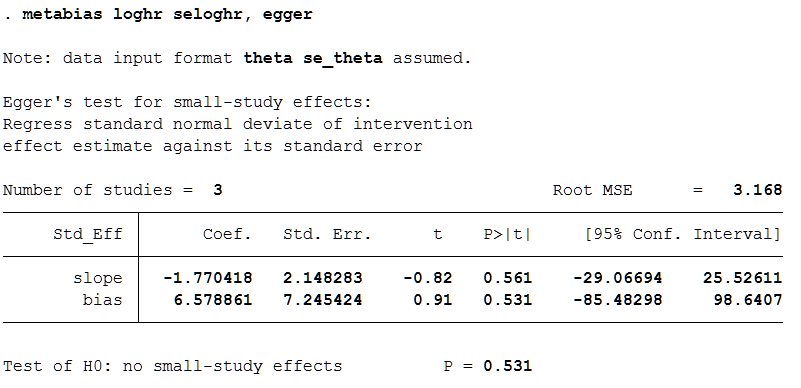

Supplement: S1 Fig — (DOC) [file pone.0145803.s002.doc]

S2 Fig.


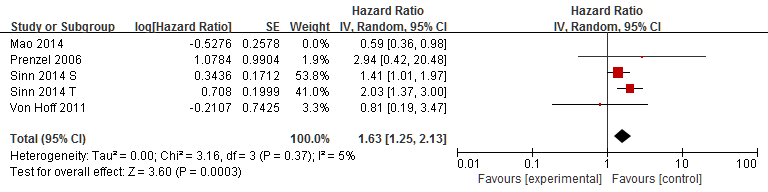


S2 Fig. Sensitivity analyses of estimate without the study of Mao.

Supplement: S2 Fig — (DOCX) [file pone.0145803.s003.docx]
