# Supplementary material for: Prognostic Value of SPARC in Patients with Pancreatic Cancer: A Systematic Review and Meta-Analysis
Source: PLoS One. 2016 Jan 5;11(1):e0145803. doi: 10.1371/journal.pone.0145803 (PMC4701416; doi:10.1371/journal.pone.0145803)
Supplement: S3 Fig — (DOCX) [file pone.0145803.s004.docx]

S3 Fig.A


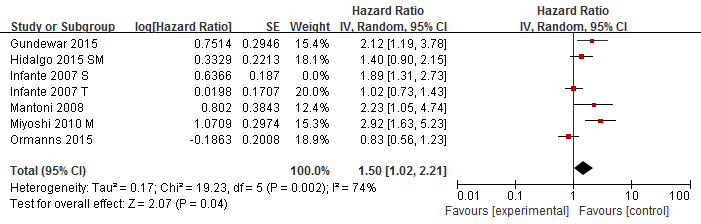


S3 Fig.B


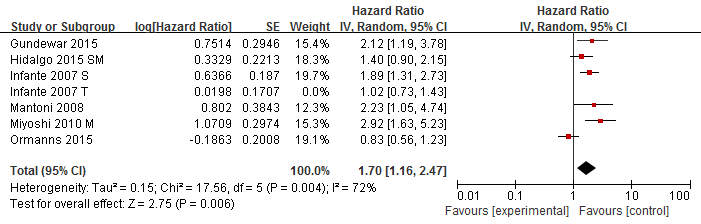


S3 Fig.C


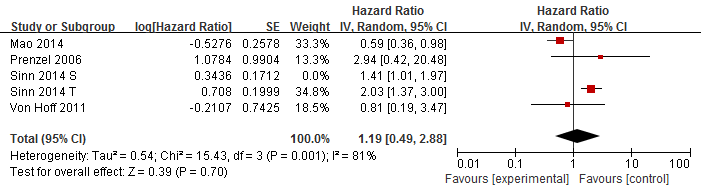


S3 Fig.D


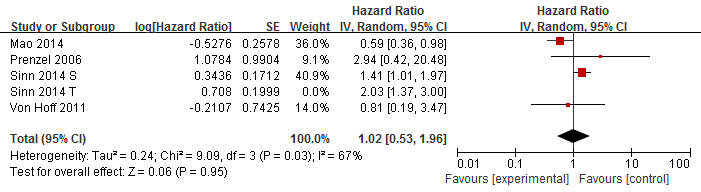


S3 Fig. Sensitivity analyses of multivariate analysis and estimate. A. and B. were about multivariate analysis; C. and D. Were about estimate.
